# The impact of financial toxicity on quality of life for survivors of primary brain tumour

**DOI:** 10.1007/s11764-025-01814-4

**Published:** 2025-04-24

**Authors:** Jasmine Luttrell, Katarzyna Lion, Mark B. Pinkham, Louisa G. Collins, Tamara Ownsworth

**Affiliations:** 1 School of Applied Psychology, Griffith University, Brisbane, Australia; 2 Department of Radiation Oncology, Princess Alexandra Hospital, Brisbane, QLD Australia; 3 School of Medicine, University of Queensland, Brisbane, QLD Australia; 4 Viertel Cancer Research Centre, Cancer Council Queensland, Brisbane, QLD Australia; 5 The Hopkins Centre, Menzies Health Institute of Queensland, Griffith University, Brisbane, Australia

**Keywords:** Brain tumour survivors, Financial toxicity, Global cognitive status, Anxiety, Quality of life

## Abstract

**Purpose:**

Despite the well-known impacts of financial hardship on cancer survivors, financial toxicity experienced in the context of brain tumour has received little attention. This study examined factors related to financial toxicity, and associations between financial toxicity, anxiety and quality of life (QoL) in this population.

**Method:**

Adults with primary brain tumour were recruited from hospital and community services as part of a telehealth psychological intervention study. They completed a telephone cognitive screener at pre-intervention, and the Comprehensive Score for Financial Toxicity-Functional Assessment of Chronic Illness Therapy (COST-FACIT), Generalised Anxiety Disorder-7 (GAD-7) and Functional Assessment of Cancer Therapy-Brain (FACT-Br) as part of a 6-week post-intervention assessment.

**Results:**

Sixty brain tumour survivors (60% female; *M* age = 47.90, *SD* = 14.47) participated. Most of the sample (57%) experienced mild to moderate financial toxicity. Individuals with high-grade glioma reported significantly higher levels of financial toxicity compared to those with benign brain tumour. Greater perceived symptoms, lower global cognitive status and higher anxiety symptoms were significantly associated with higher financial toxicity. Financial toxicity contributed significant unique variance in QoL (4.2%), controlling for covariates. Anxiety symptoms partially mediated the relationship between financial toxicity and QoL (β = 0.24, CI 0.03, 0.55).

**Conclusions:**

Higher levels of financial toxicity are related to tumour type (high-grade glioma), lower cognitive status, greater perceived symptoms and anxiety. Brain tumour survivors with higher financial toxicity are more likely to experience lower QoL, which may in part be related to greater anxiety symptoms.

**Implications for cancer survivors:**

Financial burden is associated with higher anxiety and lower quality of life for brain tumour survivors. These findings highlight the importance of early screening and access to personalised financial advice and support.

## Introduction

Primary brain tumour has been found to have detrimental impacts on survivors’ quality of life (QoL) or subjective well-being across physical, emotional, social, financial, and spiritual dimensions [1, 2]. Although the financial burden of medical treatments and disruption to employment for brain tumour survivors and family caregivers has been documented [3–5], the impact of financial toxicity on QoL has been overlooked.

Financial well-being is typically conceptualised as a dimension of QoL, reflecting individuals’ satisfaction with their financial status and ability to meet the costs of living [6]. Often overlooked in traditional measures of QoL is the related concept of “financial toxicity” which refers to the objective financial burden and distress and hardship resulting from cancer and associated treatments [7, 8]. This may relate to out-of-pocket (OOP) costs of medical care (e.g. surgery, medication, surveillance), parking, transport, carers’ inability to work or reduced hours, spending one’s savings, borrowing money or selling assets [9]. A meta-analysis of 25 cancer studies [10] identified that rates of financial toxicity measured via monetary or objective measures (e.g. OOP costs, income ratios, borrowed money, sold assets) ranged from 28 to 48%. When subjective measures (i.e. perceptions of financial toxicity and its psychological impact) of financial toxicity were used, rates varied from 16 to 73%. Cancer survivors were 2.65 times more likely to go bankrupt than age-matched individuals without cancer. Financial toxicity contributed to treatment non-adherence, reduced the capacity for choice regarding work participation and end-of-life care, and was associated with poorer QoL [10]. Other studies have identified that cancer survivors (> 6 months post-diagnosis) with higher financial toxicity have nearly three times the incidence of depression, anxiety, and psychological distress compared to those with low financial toxicity [11, 12]. Notably, no research to date has examined the impact of financial toxicity on QoL for brain tumour survivors.

In a comparison of the direct and indirect costs of seven different cancer types, brain cancer was ranked the fourth most expensive in the USA with average medical costs of US$8478 [4]. Key costs related to hospitalisation (US$4502 monthly), outpatient and emergency visits, medications, and radiology. Indirect costs such as extent of disability, working days lost due to illness, early retirement, changing one’s life to accommodate treatment costs, and mortality were estimated to constitute two-thirds of the total costs of brain tumour. For those with glioblastoma, employment rates have been found to decrease from 91 to 33% after diagnosis, with 62% of caregivers decreasing their work hours. Further, 54% of caregivers and 34% of individuals with glioblastoma had OOP costs over US$271/month [4]. A review by Pöhlmann and colleagues [13] reported that cumulative per-patient costs range from US$184,160 to 268,031 for inpatient and outpatient costs across high-grade gliomas (HGG). Further, over 10% of survivors and caregivers reported delayed or unaffordable care due to financial hardship.

Desai and colleagues [14] examined demographic factors related to financial toxicity in brain tumour survivors through four questions on a US national survey. Questions focused on the extent to which individuals felt they had to avoid or delay critical medical care because they could not afford it and whether participants’ family members had to avoid or delay their own medical care for financial reasons. Individuals with lower education attainment (e.g. high school diploma only) were significantly more likely to delay their medical care and were 10 times more likely to report that family members had to limit their own medical care to save money [14]. The influence of health-related factors (e.g. tumour type and treatment) was not examined.

Qualitative research in the USA by Bradley and colleagues [15] explored brain tumour survivors’ experience of financial hardship in the context of treatment (*n* = 20). The analysis identified four themes: impact of cancer costs, paying for medication/healthcare, strategies to offset costs, and fear/uncertainty. Despite having health insurance, participants perceived that their loss of income, high direct medical costs, and the measures required to cope with these expenses (e.g. selling their homes) had significant negative impacts for them and their families. Notably, most research to date on financial toxicity for people with brain tumour has been conducted in the USA, in which healthcare is largely provided by the private sector and costs are mainly paid for by private insurance and OOP payments [15]. Hence, individuals’ experiences of financial toxicity may differ in other countries with universal healthcare systems such as Australia. The Australian health care system comprises a mixture of public and private systems. The public system includes the government-funded Medicare program which subsidises approved medical services and medicines, although many treatments require patient co-payments. Other OOP costs for cancer care in Australia include unsubsidised care and travel and accommodation costs for treatment [16]. A systematic review of financial burden for cancer survivors and their families in Australia [17] highlighted that various groups were at risk of experiencing OOP costs and/or financial burden, including households with low income, those unemployed and people with private health insurance (i.e. due to gap payments).

Studies in the broader cancer literature have identified that financial toxicity is associated with lower psychological well-being and QoL, independent of sociodemographic and health variables [12, 17–20]. For example, in young adult cancer survivors, greater financial toxicity on the Comprehensive Score for Financial Toxicity-Functional Assessment of Chronic Illness Therapy (COST-FACIT) was associated with greater depression and anxiety symptoms, controlling for education, employment status, treatment status, and insurance satisfaction [21]. Similarly, in a sample of brain tumour survivors involved in a psychological intervention for depression, higher healthcare utilisation costs were associated with greater depressive symptoms independent of tumour grade [3]. Those with active/progressive disease had higher medication costs irrespective of tumour grade and cognitive complaints.

Overall, few studies have examined factors related to financial toxicity and associations with psychological well-being and QoL in the context of brain tumour. Based on cancer research [10, 22, 23], diverse demographic and health-related factors potentially contribute to financial toxicity and QoL. For example, Pearce and colleagues [22] identified that cancer survivors most at risk of financial toxicity were male, younger, unmarried, unemployed and had lower education and socioeconomic status. For brain tumour survivors, tumour type including benign brain tumour, lower-grade glioma (LGG) and HGG, is a major predictor of disease recurrence and symptom burden [2, 24]. Individuals with HGG typically experience greater physical and cognitive decline and require protracted treatment and specialist appointments for scans and symptom monitoring, making it more difficult to work and receive income [24]. In a systematic review of 22 studies examining health-related quality of life in adults with LGG, Rimmer and colleagues [2] found that depression, symptom duration, seizure burden, history of recurrence and tumour type (in studies with comparator HGG samples) were significantly associated with poorer QoL. Additionally, several studies have reported significant associations between cognitive impairment and QoL [25, 26]. Such impairments can affect decision-making, financial management and work participation [27, 28].

Although the relationship between depression and QoL has been well established [2, 29], brain tumour survivors also experience high rates of anxiety, which affects up to 50% and often persists post-treatment [30, 31]. Financial toxicity may impact QoL directly through restricting participation in health-promoting and social activities or indirectly through contributing to anxiety or worry regarding one’s financial concerns [17]. This in turn can reduce individuals’ ability to focus on the present and enjoy life [32]. While studies in other cancer populations show moderate to strong associations between anxiety and financial toxicity (*r* = 0.42–0.63) [19, 21], these associations are yet to be examined in brain tumour survivors.

The current study aimed to examine factors associated with financial toxicity, and the impact of financial toxicity on QoL in brain tumour survivors. It was hypothesised that greater financial toxicity would be related to tumour type (i.e. survivors with HGG would report higher financial toxicity than those with benign brain tumour or LGG), lower cognitive status, greater perceived brain tumour symptoms and greater anxiety symptoms. Further, it was hypothesised that greater financial toxicity would be significantly related to lower QoL after controlling for covariates. The potential mediating effect of anxiety on the relationship between financial toxicity and QoL was examined as a secondary aim.

## Method

### Study design and sample

Following ethical clearance from university (GUHREC/2018/389) and hospital ethics committees (HREC/QMS/43843), measure of global cognitive status, financial toxicity, anxiety, perceived brain tumour symptoms and QoL were administered as part of the Telehealth Making Sense of Brain Tumour (Tele-MAST) clinical trial [33]. Brain tumour survivors at any stage of the disease received a telephone-based cognitive screening assessment to determine eligibility prior to randomised allocation to one of two intervention conditions, the Tele-MAST program (10 sessions of psychotherapy delivered via videoconference) or standard care (telephone-based counselling from cancer support services). As relevant to the current study, financial toxicity was assessed at 6 weeks post-intervention along with anxiety and QoL as part of a health economics evaluation, as previously published [34].

Data were utilised for brain tumour survivors who participated in Tele-MAST clinical trial between 2018 and 2021 [33]. Treating health professionals screened potential participants according to the following criteria: 1) diagnosis of primary brain tumour, 2) reported at least mild distress (≥ 4 on Distress Thermometer) [35], 3) displayed sufficient cognitive and language abilities; and 4) residing in Queensland, Australia.

### Measures

#### Socio-demographic and health information

Questions relating to age, sex, education, income protection (i.e. insurance policy to cover loss of income due to illness or injury), government allowance (e.g. “JobSeeker” payments, disability support pension), relationship and employment status, and health information (e.g. tumour type, recurrence, and months since diagnosis) were included in a telephone-based survey as part of the pre-intervention assessment. Medical information including tumour type and treatment was confirmed by the referring health professional [33].

#### Global cognitive status

The Brief Test of Adult Cognition by Telephone (BTACT) [36] was administered as a brief screening tool, to obtain a global index of cognitive status. Five of the seven subtests were administered which included Digits Backward, Word List Short-Delay Recall, Backwards Counting, Category Fluency and Word List Delayed Recall. Two subtests (Attention-Switching/Reaction Time and Reasoning) were not administered due to previous experiences of administrative challenges for brain tumour survivors [37]. Participants’ age-adjusted Z-scores were derived from raw scores relative to age norms and were summed and divided by five to provide a composite score as per previous brain tumour research [38, 39].

#### Anxiety

The Generalised Anxiety Disorder- 7 (GAD- 7) [40] is a 7-item self-report scale, assessing anxiety symptomology (e.g. “feeling nervous, anxious, or on edge”) within the last 2 weeks. Items are rated on a 4-point Likert scale (0 = never, 3 = almost always) and summed to produce a total score (0–21), with higher scores indicating more severe symptoms of anxiety. A score of 5 or higher indicates clinically elevated anxiety. The GAD- 7 has sound psychometric properties and has been used frequently in brain tumour research [38, 39, 41]. Internal consistency for the current sample was good, α = 0.89.

#### Financial toxicity

The Comprehensive Score for Financial Toxicity-Functional Assessment of Chronic Illness Therapy (COST-FACIT Version 2) [11] is a 12-item self-report scale measuring objective financial burden and subjective financial distress for cancer survivors. Based on the past seven days, participants rate items on a 5-point Likert scale (0 = not at all, 4 = very much) [11]. Example items include: “My out-of-pocket medical expenses are more than I thought they would be” and “My illness has been a financial hardship to my family and me” [11]. Relevant items are reverse coded, and scores are summed to produce a total score (0–48), with lower scores indicating higher financial toxicity [42]. According to Joshi and colleagues [43], a COST-FACIT score ≥ 26 indicates “no impact”, 14–25 indicates “mild impact”, 1–13 indicates “moderate impact” and a score of 0 demonstrates “severe impact”. Good test–retest reliability (ICC of 0.80), and evidence of convergent and divergent validity have been reported in cancer samples [44], although the tool is yet to be used in brain tumour survivors. Internal consistency for the current sample was good, α = 0.80.

#### Quality of life

The Functional Assessment of Cancer Therapy-Brain (FACT-Br) [45] measures QoL (FACT-G, 27 items) and brain tumour symptoms (23 items). The FACT-G [46] is a 27-item self-report measure with four subscales assessing physical, social/family, emotional, and functional well-being that comprise a global composite. Participants rate items on a 5-point Likert scale (0 = not at all, 4 = very much) with higher scores reflecting better QoL. The FACT-G has demonstrated good reliability and validity in primary brain tumour samples [45–47]. Internal consistency in the current sample was excellent, α = 0.91.

#### Perceived brain tumour symptoms

The brain module of the FACT-Br assesses perceived brain tumour symptoms. The 23 items are rated on a 5-point Likert scale (0 = not at all, 4 = very much) and relate to cognitive, sensory, motor, and emotional functioning. Lower scores reflect greater perceived symptoms. The brain module has well established psychometric properties in the brain tumour population [45]. Internal consistency in the current sample was excellent, α = 0.92.

### Data analysis

The IBM Statistical Package for the Social Sciences (SPSS) version 29 was used for statistical analyses. After accounting for participant attrition at 6 weeks post-intervention (*n* = 22), there were no missing data or entry errors. Data were examined for assumptions of parametric analysis. Months since diagnosis was significantly skewed, which was addressed through a square root transformation. This did not alter the significance of findings and therefore relevant analyses are based on the untransformed data. One-way ANOVAs were conducted to examine categorical variables (e.g. tumour type, employment status) related to financial toxicity. Pearson’s product-moment correlations were conducted to examine associations between continuous variables and financial toxicity and identify potential covariates for the regression analysis (note:* t* tests were used for dichotomous variables such as intervention allocation). A hierarchical regression analysis was conducted to determine whether financial toxicity contributed significant variance in QoL after controlling for other relevant variables selected on the basis of univariate analyses (*p* < 0.05). The mediating effects of anxiety on the relationship between financial toxicity and QoL was examined using mediated regression analyses via the PROCESS Macro version 4.2, using 5000 bootstrapped samples [48].

## Results

### Sample characteristics

Of the 82 brain tumour survivors recruited to the Tele-MAST clinical trial, 60 (Tele-MAST = 28; standard care = 32) were retained at the 6-week post-intervention follow-up. As reported previously [33], data were not available for 22 participants for various reasons (12 discontinued the intervention or declined further assessment, 8 were unable to be contacted and 2 were deceased). Participants in the current sample were mainly female (60%) and aged 18–82 years (*M* = 47.90, *SD* = 14.47). As reported in Table 1, nearly half of the sample had HGG (45%), and the most common tumour types were glioblastoma (25%) and meningioma (23%). Time since diagnosis ranged from 0–287 months (*M* = 50.43, *SD* = 61.94).

**Table 1:** Summary of participants’ demographics and health-related characteristics (*n* = 60)

Demographic and health-related characteristics	*n(%)*	*M*	*SD*	Range
Sex				
Male	24 (40)			
Female	36 (60)			
Age (years)		47.88	15.72	18–82
Education (years)		14.44	3.03	5–25
Marital status				
Single	13(22)			
De facto (living together)	3 (5)			
Married	39(65)			
Divorced	2 (3)			
Widowed	3 (5)			
Current employment status				
Full-time	10 (17)			
Part-time/casual	10 (17)			
Student	3 (5)			
Volunteer	1 (2)			
Home duties	2 (3)			
Sickness/disability benefits	12 (20)			
Retired	10 (17)			
Unemployed	12 (20)			
Months since diagnosis		50.43	61.94	0–287
Disease status				
Initial disease	49(82)			
Recurrent disease	11 (18)			
WHO tumour grade^a^				
Grade 1	15(25)			
Grade 2	18(30)			
Grade 3	12(20)			
Grade 4	15(25)			
Tumour type				
Benign	21 (35)			
LGG	12 (20)			
HGG	27 (45)			
Tumour type				
GBM	15 (25)			
Meningioma	14 (23)			
Astrocytoma	12 (20)			
Vestibular schwannoma	7 (12)			
Oligodendroglioma	5 (8)			
Glioma (subtype unknown)	5 (8)			
Other	2 (3)			

*GBM* glioblastoma, *HGG* high-grade glioma, *LGG* lower grade glioma, *WHO* World Health Organization

### Descriptive statistics

Table 2 displays descriptive statistics on the BTACT, COST-FACIT, GAD- 7, and FACT-Br. Global cognitive status was in the “average” range for most participants (72%), although BTACT scores varied considerably (*Z* score range: − 2.76 to 1.73). Most participants (70%) were in the clinical range for anxiety (GAD- 7 score: *M* = 7.30, *SD* = 4.97). On the COST-FACIT, scores ranged from 8 to 40 (*M* = 23.92, *SD* = 8.56), with responses for 57% of the sample indicating mild to moderate financial impact of illness, whereas scores for 43% were in the range indicating “no impact”. At an individual item level, financial impacts were greatest for employment (i.e. feeling frustrated about inability to work; *M* = 1.70, *SD* = 1.62), sense of control (i.e. feel in control of financial situation; *M* = 1.87, *SD* = 1.43) and worrying about financial problems in the future (*M* = 1.92, *SD* = 1.39).

**Table 2:** Descriptive statistics for global cognitive status, anxiety, financial toxicity and quality of life in brain tumour survivors

Measures	Participants (*n* = 60) *M* (SD), *N* (%)
BTACT	− 0.33 (.957), − 2.76 to 1.73
Impaired	13 (22)
Average	43 (72)
Above average	4 (7)
GAD- 7	7.30 (4.97), 0–19
Minimal	18 (30)
Mild	23 (38)
Moderate	13 (22)
Severe	6 (10)
FACT-Br	
FACT-G	70.03 (17.32), 29–101
Brain module	59.30 (14.67), 25–91
COST-FACIT^a^	23.92 (8.56), 8–40
No impact	26 (43)
Mild impact	26 (43)
Moderate impact	8 (13)
Severe impact	0 (0)
COST-FACT items	
1. Enough money in savings for retirement	2.47 (1.37), 0–4
2. OOP expenses	2.33 (1.40), 0–4
3. Worry about financial problems in future	1.92 (1.39), 0–4
4. No choice about money I spend	2.03 (1.53), 0–4
5. Frustrated that I cannot work	1.70 (1.62), 0–4
6. Satisfied with financial situation	2.03 (1.39), 0–4
7. Able to meet monthly expenses	2.93 (1.13), 0–4
8. Feel financially stressed	2.32 (1.44), 0–4
9. Concerned about income	2.37 (1.64), 0–4
10. Cancer/treatment has reduced satisfaction with financial situation	2.05 (1.56), 0–4
11. Feel in control of financial situation	1.87 (1.43), 0–4
12. Illness has been a financial hardship	2.23 (1.44), 0–4

^a^Lower scores on the COST-FACIT indicate higher financial toxicity

### Factors associated with financial toxicity

There was a significant effect of brain tumour type on financial toxicity, *F* = 3.21,* p* = 0.048, *ηp*^*2*^ = 0.10. Pairwise comparisons indicated that survivors with HGG reported significantly higher financial toxicity (*M* = 21.66, *SD* = 7.48) than those with benign tumours (*M* = 27.57, *SD* = 8.78, *t* = 2.51, *p* = 0.044, *d* = 0.73). There was no significant difference between individuals with benign tumour and LGG (*M* = 22.58, *SD* = 9.00, *t* = 1.56, *p* = 0.13, *d* = 0.56), or LGG and HGG (*t* = 0.33, *p* = 0.74, *d* = 0.12).

As shown in Table 3, Pearson’s product-moment correlations identified that lower global cognitive status (*r* = 0.26, *p* < 0.05), greater perceived brain tumour symptoms (*r* = 0.48, *p* < 0.01) and greater anxiety symptoms (*r* = − 0.27, *p* = 0.040) were significantly associated with higher financial toxicity. Age, education and months post-diagnosis were not significantly associated with financial toxicity (*r* = − 0.05–0.12, *p* > 0.05). There were no significant differences in financial toxicity according to sex, relationship status, treatment type, number of treatments, employment status (working/not working), income protection (note: this was only reported by 3 individuals) and government allowance (see Table 4).

**Table 3:** Pearsons correlations between health-related characteristics, financial toxicity and QoL

	BTACT	GAD- 7	FACT-Br	FACT-G	COST-FACIT	Age	Education	Months since diagnosis
BTACT	-							
GAD- 7	−.21	-						
FACT-Br	.34**	−.41**	-					
FACT-G	.15	−.57***	.70***	-				
COST-FACIT	.26*	−.27*	.48***	.58***	-			
Age	.04	−.15	−.17	−.04	.11	-		
Education	.19	−.31*	.23	.18	−.05	−.27*	-	
Months since diagnosis	−.08	.22	−.24	−.26*	−.12	.26*	−.22	-

^*^p <.05;**p <.01; ***p <.001

**Table 4:** Between-group analyses of factors associated with financial toxicity (COST-FACIT) and quality of life (FACT-G)

Factor (*n*)	COST-FACIT *M* (SD)	*F* (*ɳ*_*p*_^2^)	FACT-G *M* (SD)	*F* (*ɳ*_*p*_^2^)
Sex		0.338 (.01)		0.014 (.00)
Female	23.38 (8.98)		70.25 (17.85)	
Male	24.70 (8.01)		69.71 (16.85)	
Relationship status		0.214 (.01)		1.41 (.02)
In relationship	23.53 (8.98)		71.97 (16.45)	
Single	24.62 (7.89)		66.43 (18.70)	
Total number of treatment modalities received 1 modality (8) 2 modalities (18) 3 modalities (14) 4 modalities (15)	25.18 (10.27) 24.82 (8.90) 25.28 (7.71) 20.40 (6.70)	0.841 (.06)	70.09 (14.22) 76.24 (15.08) 66.00 (21.29) 67.73 (18.64)	0.857 (.06)
Treatment modality				
Surgery		0.002 (.00)		0.110 (.00)
Yes	23.93 (8.70)		70.38 (18.75)	
No	23.81 (8.29)		68.45 (8.79)	
Chemotherapy		1.39 (.02)		0.421 (.01)
Yes	22.14 (7.52)		68.04 (18.72)	
No	24.87 (9.02)		71.10 (16.66)	
Radiotherapy		1.21 (.02)		1.32 (.02)
Yes	22.85 (7.07)		67.79 (19.36)	
No	25.30 (10.17)		72.96 (14.03)	
Gamma Knife		0.438 (.01)		0.019 (.00)
Yes	25.66 (7.38)		70.77 (11.90)	
No	23.60 (8.78)		69.90 (18.19)	
Steroids		3.04 (.05)		0.727 (.01)
Yes	22.47 (7.92)		68.58 (17.94)	
No	26.40 (9.23)		72.54 (16.26)	
Current employment status Working	25.12 (8.92)	0.681 (.04)	70.84 (17.30)	0.305 (.02)
Not working	23.88 (8.54)		70.15 (17.51)	
Income protection		0.428 (.01)		0.207 (.00)
Yes	20.00 (11.31)		75.55 (31.75)	
No	24.05 (8.55)		69.84 (17.07)	
Government allowance		1.28 (.02)		0.762 (.01)
Yes	20.17 (6.82)		64.16 (15.85)	
No	24.33 (8.69)		70.69 (17.48)	

### Multivariate analysis of the relationship between financial toxicity and quality of life

In terms of other variables of interest and potential covariates, greater perceived brain tumour symptoms (*r* = 0.70, *p* < 0.001), higher levels of anxiety (*r* = − 0.57, *p* < 0.001) and greater months since diagnosis (*r* = − 0.26, *p* = 0.047) were significantly associated with lower QoL. There were no significant associations between demographic, cognitive or health-related variables and QoL (see Tables 3 and 4). However, as reported previously [32], Tele-MAST participants reported better QoL (*M* = 75.87, *SD* = 14.14) at 6 weeks post-intervention than standard care participants (*M* = 64.94, *SD* = 18.42, *t* = 2.55, *p* = 0.013, *d* = 0.66).

A hierarchical regression analysis was conducted to determine whether financial toxicity accounted for significant variance in QoL after adjusting for other variables (see Table 5). In step 1, the combination of months since diagnosis, perceived brain tumour symptoms, anxiety and intervention allocation significantly accounted for 67% of the variance in QoL, *F* = 27.96, *p* < 0.001. In step 2, financial toxicity accounted for a further 4.2% of the variance in QoL, *F*_*chg*_ = 7.83, *p* = 0.007. Months since diagnosis (*β* = − 0.05, *t* = − 0.64, *p* = 0.523) and intervention allocation (*β* = − 0.07, *t* = − 0.86, *p* = 0.396) did not account for unique variance in the final model. Anxiety (*β* = − 0.25, *t* = 2.82, *p* = 0.007), perceived brain tumour symptoms (β = 0.52, *t* = 5.78, *p* < 0.001) and financial toxicity (*β* = 0.24, *t* = 2.80, *p* = 0.007) contributed significant unique variance in the final model, and explained 4%, 18%, and 4% of variance in QoL, respectively.

**Table 5:** Hierarchical multiple regression analysis of factors related to QoL

Variables	*R*^2^	*R*^2^*ch*	*B*	*SE*(*B*)	*β*	*t*	*Sr*^2^
Step 1	.67	.67					
Months since diagnosis			−.01	.02	−.05	− 0.58	.05
Tele-MAST allocation			− 4.24	2.96	−.12	− 1.43	.11
Perceived symptoms			.75	.10	.63	7.30***	.56
Anxiety			−.86	.33	−.25	− 2.64*	.20
Step 2	.71	.04					
Months since diagnosis			−.01	.02	−.05	0.64	.05
Tele-MAST allocation			− 2.45	2.86	−.07	0.86	.06
Perceived symptoms			.62	.11	.52	5.78***	.42
Anxiety			−.87	.31	−.25	2.82**	.21
Financial toxicity			.49	.17	.24	2.80**	.20

^*^p <.05; **p <.01; ***p <.001

In relation to the secondary aim, there was a significant indirect effect of financial toxicity on QoL through anxiety (β = 0.24, *SE* = 0.13, lower CI 0.03, upper CI 0.55). The direct effect of financial toxicity on QoL was also significant (β = 0.94, SE = 0.19, lower CI 0.55, upper CI 1.32). Therefore, the relationship between financial toxicity and QoL was partially mediated by anxiety symptoms.

## Discussion

This study aimed to examine factors related to financial toxicity and the association between financial toxicity and QoL in brain tumour survivors. Based on interpretation guidelines for cancer survivors by Joshi and colleagues [43], just over half of our brain tumour sample (57%) experienced at least mild financial impact of illness, although no survivors rated the impact as severe. As hypothesised, levels of financial toxicity for survivors with HGG were higher than for those with benign brain tumour, although did not differ from those with LGG. Further, lower global cognitive status, greater perceived brain tumour symptoms and anxiety were related to higher financial toxicity. Higher levels of financial toxicity were independently related to poorer QoL, and this relationship was partly mediated by anxiety symptoms.

The higher levels of financial toxicity evident for survivors with HGG relative to those with benign brain tumour may be related to the costs associated with more prolonged treatment and intensive surveillance, in addition to reduced income due to functional decline and greater caregiving needs. Chang and colleagues [49] identified that, in addition to higher monthly OOP costs, the average household income loss was higher for HGG than for other cancers, which may be due to illness-related disability impacting employment for both brain tumour survivors and their family caregivers [49]. In the current study, there was no significant difference in levels of financial toxicity between survivors with LGG and HGG. Although caution is needed with interpretation due to the small number of survivors with LGG (*n* = 12), individuals with LGG are typically younger adults of working age between 35 and 44 years [50]. They may not have accumulated savings to cover medical costs which can also be significant for LGG [13]. Research has highlighted that financial strain associated with employment changes (e.g. going part-time) can make it more difficult to earn sufficient income, leading to increased reliance on family members for financial support [51]. In contrast to previous cancer research [17, 22], younger age was not associated with financial toxicity, which may be related to the finding that younger participants in the current study were typically highly educated (see Table 3), which in turn is often related to socio-economic status.

Higher financial toxicity was also related to lower global cognitive status and greater perceived symptom burden and anxiety. Such findings extend upon those of Boele and colleagues [3] who identified that higher healthcare utilisation and medication costs were associated with greater depression and disease status (i.e. active/progressive disease), respectively. Cognitive impairment and brain tumour symptoms can arise from damage to brain structures as well as treatment side effects [39, 52], impacting independence, finance management and the capacity to return to work and earn an income [13]. The finding that anxiety was positively associated with financial toxicity and negatively associated with QoL in brain tumour survivors is consistent with previous cancer research [31, 32]. As measured by the COST-FACIT, financial strain from treatment costs (e.g. hospital stays, medications), loss of income and worrying about one’s family’s financial position in the future is likely to exacerbate anxiety in the context of other illness-related stressors (e.g. threat to life, functional decline).

Extending on previous research [14, 19, 20], financial toxicity was found to influence QoL both directly and indirectly through anxiety. Financial difficulties arising from brain tumour can lead to people foregoing necessities such as food and clothing and create barriers to accessing support and treatments such as medication, which can exacerbate disease symptoms [53]. Financial strain may also limit social activities, hobbies, and other meaningful activities, reducing life satisfaction even if physical and mental health are stable [38, 54]. As suggested by the current findings, financial toxicity may contribute to individuals’ anxiety concerning the ability to afford costs of living and their family’s security in the long-term, which in turn reduces QoL.

### Implications

Our findings highlight that survivors with HGG and those with lower global cognitive status and greater brain tumour symptoms are at higher risk of experiencing financial toxicity. Hence, screening for financial toxicity is a priority in supportive care for these individuals. Although there is evidence that psychological support interventions can reduce anxiety and improve QoL [33], interdisciplinary approaches that target the reduction of financial toxicity in the context of brain tumour are needed [9, 55]. If not identified and managed early, financial burden can escalate rapidly and impact access to ongoing treatment [9]. Financial navigation programs that assist with advanced planning for treatment costs and employment changes may support brain tumour survivors to consider their options, be prepared and minimise the stress associated with protracted expenses [9, 55, 56]. Sadigh and colleagues [56] piloted a financial navigation program for brain tumour survivors alongside their treatment. Navigators determined individuals’ financial concerns or needs, provided tailored assistance (e.g. access to health insurance coverage, debt relief, cost of living and disability applications) and then monitored individuals through monthly contact for a minimum of 6 months. Despite low accrual (11%) and retention (42%) the findings broadly supported the feasibility of a financial navigation program for those interested in accessing such support.

Researchers have highlighted the need for financial care standards for brain tumour, to enable prioritisation of resource allocation to those most at risk of financial toxicity. This can be undertaken by creating risk profiles through financial-related outcome measures (FROMs) [57] to identify and monitor financial toxicity throughout the illness, revealing peak periods of finance-related distress to inform timely interventions [57]. Further research utilising FROMs is recommended to understand the timing and impact of financial toxicity for brain tumour survivors.

### Limitations

This study is the first to examine the association between financial toxicity and QoL in the context of brain tumour. Nonetheless, some limitations may impact the interpretation of findings and their broader applicability. Due to the cross-sectional design, caution is needed in inferring the direction of relationships among financial toxicity, anxiety and QoL, particularly given the conceptual overlap (e.g. worry regarding one’s finances may contribute to generalised anxiety and vice versa). In future research, longitudinal designs are recommended with early and repeated measurement of financial toxicity to determine the predictive value for changes in anxiety and QoL. Such research could also inform the optimal timing of interventions targeting financial-related distress.

In terms of sampling, there was considerable heterogeneity in terms of brain tumour characteristics and varying time since diagnosis (0–287 months), all of which could impact the salience of financial toxicity over the survivorship trajectory as well as associations with QOL. All participants had previously reported at least mild distress and had completed a psychological support program through Tele-MAST or cancer support services (standard care). Tele-MAST was associated with significant improvements in anxiety and QoL at both post-intervention and 6 weeks post-intervention relative to standard care [33]. Although intervention allocation was controlled for in the multivariate analysis of factors related to QoL, measures of financial toxicity, anxiety and QoL were administered after individuals had received psychological support. More generally, the current sample reflects individuals who sought and received psychological support, which may not be representative of the broader population of brain tumour survivors. As a further limitation, the BTACT was administered as part of the initial screening, which was several months before assessment of financial toxicity, anxiety and QoL. Some participants may have experienced cognitive decline throughout this period. In future, cognitive testing should occur concurrent with self-report measures to ensure data reflect participants’ current cognitive status.

In future research, it would be beneficial to examine factors that buffer or protect individuals from the effects of financial toxicity, such as social support, coping strategies and resilience. For example, in breast cancer research, survivors with higher family resilience experienced lower levels of financial toxicity [58]. Resilient families are often better at problem-solving and adapting to new challenges, such as navigating financial issues or adjusting budgets in response to medical expenses, reducing pressure on the cancer survivor. More generally, along with the development of risk profiles using FROMs, the role of personal and social resources in buffering the effects of financial toxicity on QoL in brain tumour survivors represents an important area for future research.

## Conclusion

This study highlighted various risk factors for financial toxicity in brain tumour survivors and identified that higher financial toxicity is related to poorer QoL, partly through greater anxiety symptoms. These findings emphasise the importance of early and regular screening along the illness trajectory and management of financial toxicity as a potential avenue for improving QoL. Longitudinal research is needed to monitor financial toxicity throughout the illness to identify peak periods of finance-related distress to inform timely interventions.

## Data Availability

The data that support the findings of this study are available from the corresponding author, but restrictions apply to the availability of these data and so are not publicly available. The data are, however, available from the authors upon reasonable request and with the permission of Griffith University.
